# Mesoglycan attenuates VSMC proliferation through activation of AMP-activated protein kinase and mTOR

**DOI:** 10.1186/s40885-016-0037-x

**Published:** 2016-01-18

**Authors:** Kyung Young Lee, Dong Hyup Lee, Hyoung Chul Choi

**Affiliations:** 1 Department of Pharmacology, College of Medicine, Yeungnam University, 170 Hyunchung-Ro, Nam-Gu, Daegu, 42415 Republic of Korea; 2 Department of Thoracic and Cardiovascular Surgery, College of Medicine, Yeungnam University, 170 Hyunchung-Ro, Nam-Gu, Daegu, 42415 Republic of Korea; 3 Smart-aging Convergence Research Center, College of Medicine, Yeungnam University, 170 Hyunchung-Ro, Daegu, 42125 Republic of Korea

**Keywords:** Mesoglycan, AMPK, VSMC, mTOR, Proliferation

## Abstract

**Background:**

Vascular smooth muscle cells (VSMC) proliferation contributes significantly to intimal thickening in atherosclerosis and restenosis diseases. Platelet derived growth factor (PDGF) has been implicated in VSMC proliferation though the activation of multiple growth-promoting signals. Mesoglycan, a natural glycosaminoglycans preparation, is reported to show vascular protective effect. However, the mechanisms by which mesoglycan inhibits proliferation of VSMC are not fully understood. Here, we investigated whether mesoglycan exert therapeutic effect via AMP-activated protein kinase (AMPK) and its underlying mechanism.

**Methods:**

We cultured VSMC with increasing doses of mesoglycan. AMPK activation was measured by western blot analysis and cell proliferation was measured by flow cytometry.

**Results:**

Mesoglycan dose- and time- dependently increased the phosphorylation of AMPK (Thr^172^) and its upstream target, LKB1 (Ser^428^) and its downstream, ACC (Ser^79^) in VSMCs. Mesoglycan also blocked the PDGF-stimulated cell cycle progression through the G_0_/G_1_ arrest. AMPK DNα1, AMPK DNα2 or AMPK siRNA reduced the mesoglycan-mediated inhibition of VSMC proliferation. AMPK signaling activated by mesoglycan regulates mTOR phosphorylation which closely related to cell proliferation.

**Conclusion:**

These data suggest that mesoglycan-induced AMPK activation suppress the VSMC proliferation via mTOR-dependent mechanism and mesoglycan may have beneficial effects on vascular proliferative disorders such as atherosclerosis.

## Background

The proliferation of vascular smooth muscle cells (VSMC) is an important pathogenic factor in vascular disease such as atherosclerosis and hypertension [1–3]. Proliferation of VSMCs is activated by various growth factors, cytokines, and extracellular matrix components. Ultimately, mammalian target of rapamycin (mTOR) activity regulates both cell cycle progression and cell growth [4–6]. The vascular smooth muscle cell proliferation, elevated mTOR phosphorylation, an indicator of mTOR intrinsic catalytic activity [7]. Thus, inhibition of VSMC proliferation may reduce the development of vascular proliferative disease.

AMP-activated protein kinase (AMPK) is a serine/threonine protein kinase, which serves as an energy sensor in all eukaryotic cell types. Published studies indicate that AMPK activation strongly suppresses cell proliferation in non-malignant cells as well as in tumor cells [8, 9]. The importance of AMPK in cardiovascular functions is best demonstrated by recent studies showing that widely used drugs, including statins, metformin and rosiglitazone, execute cardiovascular protective effects at least partly through the activation of AMPK [10–12]. Among these, research on the protective actions of and the regulation of energy metabolism by AMPK in the vascular smooth muscle is becoming an important area. Therefore, we focus on the effect of AMPK activation on cellular proliferation and discuss the possibility that AMPK might be a therapeutic target for proliferative disorders such as atherosclerosis and cancers.

Mesoglycan is a mucopolysaccharide complex that is extracted from calf aorta. Aortic glycosaminoglycans and mucopolysaccharides such as mesoglycan are used to treat diseases of blood vessel homeostasis, blood clotting, atherogenesis, and atherosclerosis [13, 14]. Mesoglycan is composed of heparan sulfate, dermatan sulfate, heparin and minimal quantities of chondroitin sulfate [15]. One of the components that heparan sulfate proteoglycan is reported to inhibit the growth of VSMC in culture [16, 17]. Natural glycosaminoglycans such as heparin are also known to inhibit VSMC proliferation in vitro in tissue culture and in vivo in animal models [18–20]. Therefore, we hypothesized that mesoglycan may has the anti-proliferative effects in VSMCs.

Recent study has demonstrated that glucosamine suppress adipocyte differentiation and adipogenesis through the up-regulation of AMPK pathway [21]. Hence, the purpose of this study is to determine whether mesoglycan can induce AMPK activation in VSMCs and to determine whether mesoglycan-induced AMPK activation inhibits cell proliferation via an mTOR-dependent mechanism.

## Methods

### Materials

Mesoglycan was kindly provided from Chodang Pham. CO.. Dulbecco’s modified eagle medium (DMEM) and fetal bovin serum (FBS) were purchased from Thermo Scientific (Logan, UT, U.S.A.). Pro-prep protein extract buffer was purchased from Intron Biotechnology (Sungnam, Korea). Antibodies against LKB1, phospho-LKB1 (Ser^428^), AMPK, phospho-AMPK (Thr^172^), phospho-ACC (Ser^79^), mTOR, phospho-mTOR (Ser^2448^), Bcl-2, Bax, phospho-p70S6K (Thr^389^) and phospho-4EBP1 (Ser^65^) were purchased from Cell Signaling Technology (Beverly, MA, U.S.A.). A monoclonal antibody against β-actin was purchased from Sigma-Aldrich (St. Louis, MO). Compound C, AMPK inhibitor, was provided by Calbiochem (La Jolla, CA, U.S.A.). Human Platelet-Derived Growth Factor BB was purchased from Cell Signaling Technology (Beverly, MA, U.S.A.). AMPK siRNA, Raptor siRNA, Control siRNA, p53, p27, p21, PCNA and Cytochrome C were purchased from Santa Cruz Biotechnology (Santa Cruz, CA, U.S.A.).

### Cell culture

Sprague–Dawley rats (SD rats) were anesthetized with pentobarbital (50 mg/kg). Vascular smooth muscle cells (VSMCs) were isolated from thoracic aorta and the connective tissue was removed. Aortic VSMCs were grown in DMEM with 10 % FBS and 1 % antibiotic (penicillin 10000 U/ml). It was processed using a 1 mm chop setting in a 10 cm culture dish, and cultured with 50 % FBS-DMEM with 1 % antibiotics and incubated in a CO_2_ incubator (95 % CO_2_ air, 37 °C). We used VSMCs from 6 to 8 passages at 70–90 % confluence in 10 cm dishes, and cell growth was arrested by incubation of the cells in serum-free DMEM for 24 h prior to use.

### Western blot analysis

Whole cell extracts were prepared by lysing the cells in pro-prep protein extract buffer. The protein concentration was quantified with protein assay reagent from Bio-Rad (Hercules, CA, U.S.A.). Equal amounts of protein were mixed with Laemmli Sample Buffer (Bio-Rad) and heated for 5 min at 100 °C before loading. Total protein samples (30 μg) were subjected to 10 % SDS-polyacrylamide gel electrophoresis (SDS-PAGE) for 1 h 30 min at 100–120 V. The separated proteins were electrophoretically transferred onto a PVDF membrane for 1 h 20 min at 100 V using SD Semi-dry Transfer Cell. The membranes were blocked with 5 % non-fat milk in PBS containing 0.05 % Tween 20 (PBS-T) for 1 h at room temperature. The membranes were then incubated with the primary antibodies at a dilution of 1:1000 by overnight at 4 °C in PBST. The membranes were then washed with four changes of wash buffer (0.05 % Tween 20 in PBS) and incubated for 1 h at room temperature in PBS containing anti-rabbit(Stress-gen, Ann Arbor, MI, USA) and anti-mouse IgG (Sigma, St. Louis, MO, USA) antibodies. Finally, after three more rinses with wash buffer, the membranes were exposed to ECL and ECL Plus western blot analysis detection reagents.

### Transfection of siRNA

Transfection of VSMCs with siRNA was performed using lipofectamine-2000 reagent, according to the manufacturer’s instructions. Aliquots of 1 × 10^4^ cells were plated on 6 well on the day before the transfection and grown to about 70 % confluence. The cells were then transfected with 10 μM AMPK siRNA (Santa Cruz Biotechnology Inc., Santa Cruz, CA) + 100 pmol of Lipofectamine for 6 h in Opti-MEM®I reduced serum medium (Invitrogen, Carlsbad, CA, USA). Following an incubation period of 48 h, the AMPK protein level was measured using western blot analysis, and cell proliferation was analyzed using the MTT assay.

### Adenoviral transduction

Adenoviruses expressing the control gene GFP, the dominant-negative isoform of the α1 and α2 subunits of AMPK (AMPK DNα1 and DNα2) were amplified in AD293 cells using standard methodologies. The transductions were carried out in VSMC in sereum-fress DMEM for 6 h.

### Flow cytometric analysis for apoptosis and cell cycle

Apoptosis was examined by Annexin V-fluorescein isothiocyanate (FITC) staining (BD Biosciences, San Jose, CA, U.S.A.) according to the manufacturer’s instructions. Cells were seeded on 6-well plates and incubated for 2 days. Cells were treated with mesoglycan (0.1 μg/ml) for 24 h. The FITC fluorescence intensity of 10,000 cells was measured using a Becton-Dickinson FACS Caliber flow cytometer (BD Biosciences). Cell cycle profiles were analyzed by propidium iodide (PI) staining. A minimum of 10,000 cells in each sample was detected according to intracellular PI fluorescence intensity by flow cytometry, and cell cycle was analyzed by Cell Quest software (BD Biosciences).

### Fluorescence intensity measurements

Cells were washed and then maintained in complete medium. After detachment from dishes with 50 mM EDTA, the cells were centrifuged at 3000 rpm for 10 min and resuspended in PBS containing 2 % bovine serum albumin. After labeling, cells were washed once in PBS, fixed in 4 % paraformaldehyde, and analyzed on a flow cytometer. For each sample, 1000 cells were analyzed, and the results were expressed as geometric mean fluorescence.

### Immunofluorescence analysis

VSMCs were seeded on coverslips in 35 mm glass bottom dishes, fixed in 4 % formaldehyde, and permeabilized with 0.2 % Triton X-100. The p-mTOR primary antibody was used at 1:100 (Santa Cruz Biotechnology) and incubated with cells overnight at 4 °C. Rabbit FITC secondary antibody (Invitrogen) was used at 1:100 and incubated with cells for 1 h at room temperature. Fixed and immunofluorescently stained cells were imaged using a Leica confocal microscope (Bannockburn, IL, USA).

#### Cell proliferation assay

VSMCs were seeded on 24-well plates at 1 × 10^4^ cells per well in DMEM supplemented with 10 % FBS. After different treatments, 50 μl of 1 mg/ml MTT solution was added to each well (0.1 mg/well) and incubated for 4 h. The supernatants were aspirated, and the formazan crystals in each well were solubilized with 200 μl dimethyl sulfoxide (DMSO). An aliquot of this solution (100 μl) was placed in 96-well plates. Cell proliferation was assessed by measuring the absorbance at 570 nm using a microplate reader.

### Statistical analysis

Results are expressed as mean ± SEM from at least three independent experiments. Differences between data sets were assessed by one-way analysis of variance (ANOVA) or Bonferroni's test.

## Results

### Mesoglycan and heparin increased phosphorylation of LKB1, AMPK and ACC in VSMCs

**Fig. 1:**
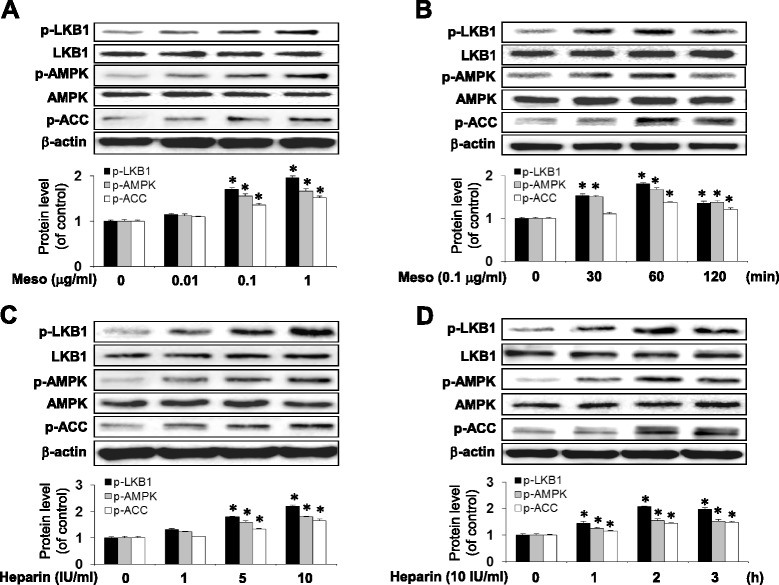
Mesoglycan and Heparin induce AMPK activation in VSMC. Cells were treated with the indicated concentration of mesoglycan for 1 h (**a**) or for the indicated periods (**b**). Protein expression of p-LKB1, p-AMPK, and p-ACC were determined by western blot analysis. VSMCs were treated with the indicated concentration of heparin for 2 h (**c**) or for the indicated periods (**d**). Protein expression of p-LKB1, p-AMPK, and p-ACC were determined by western blot analysis. Representative results from three independent experiments were shown. *p <0.05 versus the control

Mesoglycan (0.01–1 μg/ml for 1 h) produced a dose-dependent increase in phosphorylation of LKB1 at Ser^428^, AMPK at Thr^172^ and of ACC at Ser^79^ in VSMCs (Fig. 1a). Compared with control, mesoglycan (0.1 μg/ml, 1 h) caused a drastic increase in phosphorylation of LKB1 (Ser^428^), AMPK (Thr^172^) and ACC (Ser^79^) (Fig. 1b).

We next examined whether heparin was correlated with AMPK activity. Heparin (1–10 IU/ml for 2 h) produced a dose-dependent increase in phosphorylation of LKB1 at Ser^428^, AMPK at Thr^172^ and of ACC at Ser^79^ in VSMCs (Fig. 1c). Compared with control, heparin (10 IU/ml, 2 h) caused a significant increase in phosphorylation of LKB1 (Ser^428^), AMPK (Thr^172^) and ACC (Ser^79^) (Fig. 1d). These data suggest that mesoglycan and heparin induced the dose-and time-dependent phosphorylation of LKB1, AMPK and ACC.

### PDGF-induced cell cycle progression in G_2_/M phase is reversed by mesoglycan

**Fig. 2:**
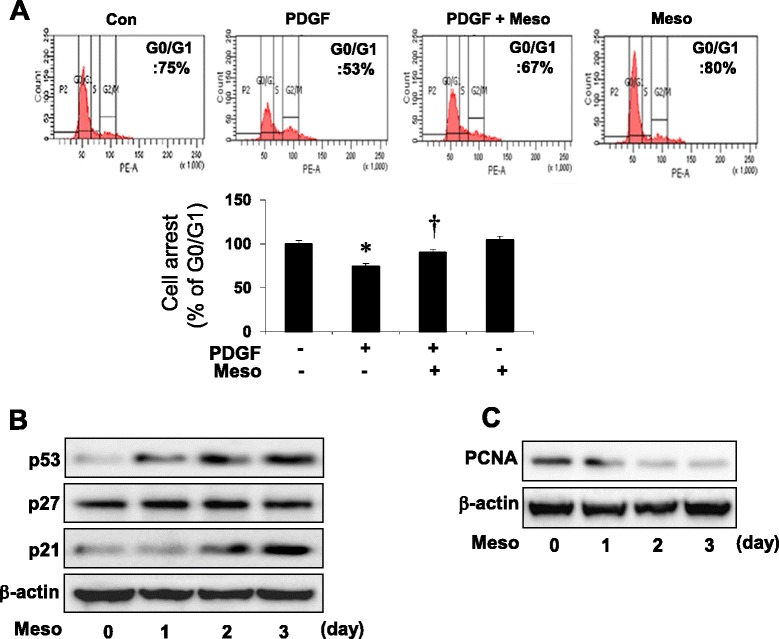
Mesoglycan-induced expressions of p53 and p21 inhibits cell cycle progression. Cells were treated with mesoglycan (0.1 μg/ml) or mesoglycan plus PDGF (10 ng/ml), and treated with PDGF for 24 h. Cell cycle analysis was assessed by PI staining and the percentage of cells in G_0_/G_1_ phase, S phase. Cells were detached by trypsinization and studied with flow cytometry (**a**). Cells were treated with mesoglycan for the indicated periods of time. Protein expressions of p53, p21, p27 and PCNA were determined by western blot analysis (**b**, **c**). *p <0.05 versus the control, ^†^ *p* <0.05 versus PDGF. Representative results from three independent experiments are shown

We examined the effect of mesoglycan on cell cycle progression using flow cytometric analysis with PI staining. After serum deprivation for 48 h, growth-arrested VSMC were treated with mesoglycan (0.1 μg/ml) and PDGF (10 ng/ml). Compared with PDGF-treated cells, mesoglycan increased the number of cells in the G_0_/G_1_ phase (Fig. 2a).

We then examined the effect of mesoglycan on the expression of p53, a key regulator of the cell cycle, as well as p21 and p27, downstream targets of p53. Mesoglycan increased p53 and p21 expression in a time-dependent manner, whereas the levels of p27 were not changed (Fig. 2b). Next, we examined the effect of mesoglycan on the expression of proliferating cell nuclear antigen (PCNA). Mesoglycan decreased PCNA expression in a time-dependent manner (Fig. 2c). These data suggest that mesoglycan blocks VSMC proliferation by increasing G_0_/G_1_ arrest and decreasing G_2_/M phase.

#### Mesoglycan-induced expression of p53 and p21 did not affect the apoptotic pathway in VSMCs

**Fig. 3:**
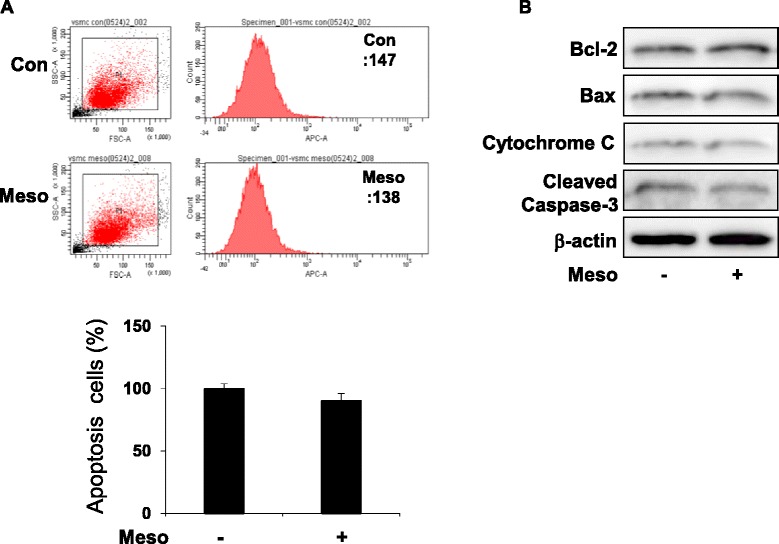
Mesoglycan does not influence apoptotic signaling pathways. Cells were treated with mesoglycan (0.1 μg/ml) for 48 h. Apoptosis was assessed by Annexin V-fluorescein isothiocyanate (FITC) staining by flow cytometric analysis and the percentage of apoptotic cells was then determined (**a**). Protein expressions of Bcl-2, Bax, cytochrome C and cleaved Caspase-3 were determined by western blot analysis (**b**). Representative results from three independent experiments are shown

The reduction in cell number induced by mesoglycan could result from either an increase in cell death or inhibition of proliferation. Thus, we examined whether mesoglycan could induce apoptosis. Apoptosis was measured by flow cytometric analysis with annexin V staining after mesoglycan treatment. Compared with control, mesoglycan-treated cells induced non-apoptotic cell death (Fig. 3a).

We then examined the effect of mesoglycan on the expression of the apoptotic pathway. There was no change in the protein levels of Bcl-2, Bax, cytochrome C and cleaved caspase-3 (Fig. 3b). These data suggest that the reduction in cell number induced by mesoglycan is not apoptotic cell death.

### Mesoglycan inhibited mTOR signaling through AMPK activation in VSMC

**Fig. 4:**
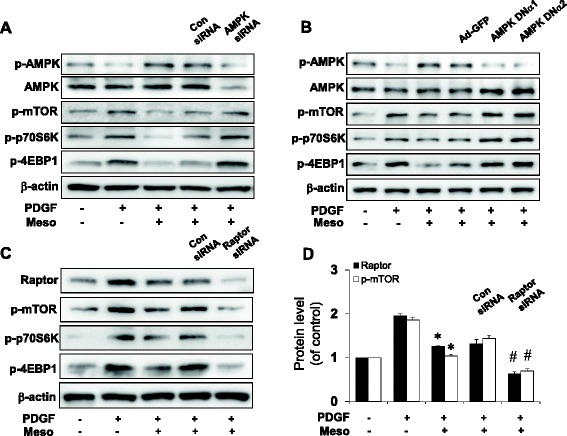
AMPK signaling regulates expressions of p-mTOR and its downstream. VSMCs were stimulated with PDGF for 24 h and then transfected with control (Con) siRNA or AMPK siRNA in the presence of mesoglycan. Protein expression of p-AMPK, AMPK, p-mTOR, p-p70S6K and p-4EBP1 were determined by western blot analysis (**a**). VSMCs were transfected with adenovirus-GFP, AMPK DNα1 and AMPK DNα2 for 6 h and then incubated for 48 h. The levels of p-AMPK, AMPK, p-mTOR, p-p70S6K and p-4EBP1 were determined by western blot analysis (**b**). VSMCs were stimulated with PDGF for 24 h and then transfected with control (Con) siRNA or Raptor siRNA in the presence of mesoglycan. VSMCs were subjected to western blotting to determine the level of Raptor, p-mTOR, p-70S6K and p-4EBP1 proteins (**c**). Densitometric analysis of Raptor and p-mTOR expressions in 4C (**d**). *p ≤0.05 versus PDGF, ^#^p ≤0.05 versus PDGF ± Meso. Representative results from three independent experiments are shown

mTOR protein kinase has the potential to regulate the G_1_ phase of the cell cycle and to stimulate cell proliferation [22]. We examined whether mTOR signaling is involved in mesoglycan-induced AMPK pathway. As shown in Fig. 4a, AMPK siRNA transfection significantly inhibited p-AMPK and total AMPK. In addition, phosphorylation of mTOR, p70S6K and 4EBP1 increased. Continuously, the adenovirus-mediated overexpression system was used to determine whether mesoglycan-induced AMPK activation could inhibit mTOR pathway. Compared to the GFP controls, transduction of AMPK DNα1 and AMPK DNα2 prevented the increases in phosphorylation of the AMPK (Fig. 4b). Additionally, as shown in Fig. 4c, we examined the effects of raptor siRNA on mesoglycan-inhibited mTOR signaling. These data suggested that the mesoglycan/AMPK pathway negatively regulates mTOR phosphorylation in VSMCs.

### Inhibition of AMPK activation increased mTOR phosphorylation in VSMC

**Fig. 5:**
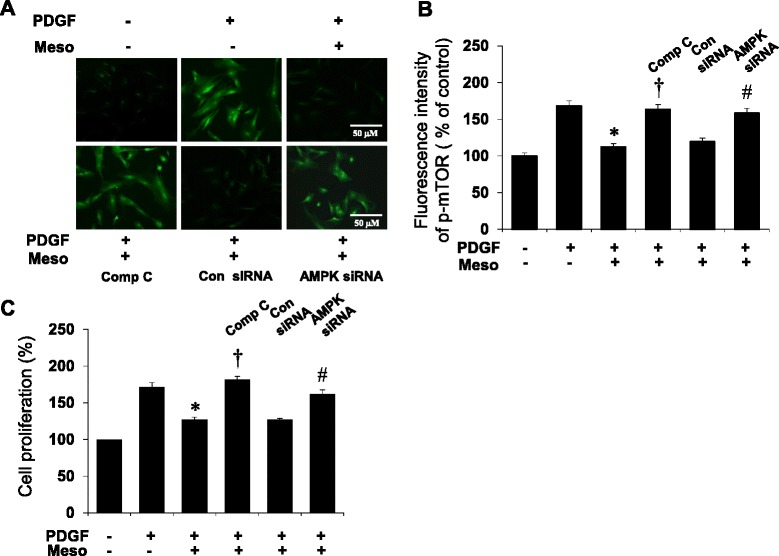
Localization and flow cytometric analysis of p-mTOR proteins and cell proliferation regulated by mesoglycan in VSMCs. Cells are stained with phosphorylated mTOR (green). All photographs are taken under confocal laser-scanning microscopy. VSMCs treated with compound C and AMPK siRNA show more mTOR phosphorylation (**a**). Flow cytometric intensity of p-mTOR protein in VSMCs. p-mTOR level was detected with p-mTOR antibody and the results were analyzed by FACS. Fluorescence intensity is geometric mean fluorescence. Each percentage is based on 1000 cells (**b**). Cells were stimulated with PDGF for 24 h and then treated with compound C (10 μM) or transfected with AMPK siRNA in the presence of mesoglycan. Cell proliferation was determined by the MTT assay (**c**). Representative results from three independent experiments were shown. *p <0.05 versus PDGF, ^†^ *p* <0.05 versus PDGF + Meso, ^#^ *p* <0.05 versus control siRNA

To further test whether mesoglycan-induced AMPK activity negatively regulates mTOR phosphorylation, cultured cells were treated with compound C and transfected with AMPK siRNA and then stained with p-mTOR specific antibodies. Immunofluorescence with the p-mTOR antibodies revealed changes in mTOR phosphorylation in compound C-treated and AMPK siRNA transfected cells compared to PDGF plus mesoglycan (Fig. 5a). In the next set of experiments, we examined this in flow cytometry studies. This analysis showed that the level of p-mTOR decreased approximately 30 % in cells incubated with mesoglycan compared to that in PDGF alone treatment (Fig. 5b).

We then examined the effect of mesoglycan on the PDGF induced VSMC proliferation. Mesoglycan decreased VSMC proliferation stimulated with PDGF. Compound C, a specific inhibitor of AMPK, reduced the mesoglycan-mediated inhibition of VSMC proliferation. Genetic inhibition of AMPK with siRNA also restored the mesoglycan induced anti-proliferative effect (Fig. 5c). These data suggest that AMPK signaling activated by mesoglycan regulates mTOR phosphorylation related to cell proliferation.

## Discussion

Mesoglycan has been shown to decrease capillary permeability to enhance systemic fibrinolysis in humans and to prevent venous thrombus formation in experimental and clinical settings [23]. But the exact mechanisms underlying the proliferation by mesoglycan in VSMC are still unknown. Metabolic cardiovascular disease is closely regulated through the mTOR signaling pathways [24, 25]. Activation of pathways of mTOR also may promote tumor growth [26] and increase the activation of imflammatory cell pathways [27, 28] that may negatively impact the cardiovascular system.

The main purpose of the present study was to demonstrate the anti-proliferative mechanism of mesoglycan in VSMC, specifically focused on the AMPK activation. Also, the exact mechanisms underlying the anti-proliferation by mesoglycan-induced AMPK activation in VSMC are still unknown.

In the present study, we first demonstrated that mesoglycan and heparin activated AMPK by phosphorylating AMPK (Thr^172^) in VSMC (Fig. 1). Mesoglycan also increased phosphorylation of LKB1, an upstream substrate of AMPK (Ser^428^), and phosphorylation of ACC, a downstream substrate of AMPK (Ser^79^). Mesoglycan-induced AMPK activation has an attractive characteristic, because AMPK is reported to mediate beneficial and bio-protective effects of metformin [29] and adiponectin [30]. AMPK is a serine/threonine protein kinase, which serves as an energy sensor in eukaryotic cells. Several studies have revealed that AMPK activation suppresses cell proliferation in normal cells as well as in tumor cells [31].

In the current study, we found that the anti-proliferative effect of mesoglycan might be in part via an AMPK-p53-p21 signaling pathway. The mechanism of growth suppression by mesoglycan is there G_0_/G_1_ cell cycle arrest (Fig. 2). These effects of AMPK are mediated through multiple mechanisms including cell cycle regulation and inhibition of protein synthesis. AMPK activation by AICAR or constitutively activated AMPK induces a G_0_/G_1_ cell cycle arrest via AMPK-dependent phosphorylation of p53 in human VSMCs [32].

Mesoglycan-induced expression of p53 and p21 does not change the apoptotic pathway in VSMCs (Fig. 3). The reduction in cell number induced by mesoglycan not increase in cell death but inhibition of proliferation.

Furthermore, several studies revealed that endothelial cells cultured from fetal bovine pulmonary arteries produce a basement membrane heparan sulfate proteoglycan that is a potent inhibitor of smooth muscle proliferation [33, 34]. In this regard, we examined whether mesoglycan can impede VSMC proliferation through activation of AMPK, because pharmacologic and genetic inhibition of AMPK restored the mesoglycan-mediated inhibition of VSMC proliferation. AMPK inhibition by AMPK siRNA prevented mesoglycan-induced phosphorylation of AMPK. AMPK inhibition by adenoviral infection encoding dominant-negative form of AMPK exhibited the increases in p-AMPK. These results confirmed that anti-proliferative effect of mesoglycan is mediated in part by an AMPK signaling pathway (Fig. 4).

In the cardiovascular and metabolic systems, mTOR and its multi-protein complexes of TORC1 and TORC2 regulate insulin release, cell growth and cardiomyocyte proliferation [35]. Our findings suggested that mesoglycan inhibited mTOR signaling and cell proliferation through AMPK pathway in VSMCs (Fig. 5).

Taken together, inhibition of mTOR signaling by mesoglycan-induced AMPK activation could induce the anti-proliferative mechanism of mesoglycan in VSMC.

## Conclusion

In this study, mesoglycan-induced AMPK activation inhibited the PDGF-induced VSMC proliferation via an mTOR-dependent mechanism. Our observations also indicate that mesoglycan-induced AMPK activation may have beneficial effects on vascular proliferative disorders such as atherosclerosis.

## Abbreviations

AMPK: AMP-activated protein kinase
mTOR: Mammalian target of rapamycin
PDGF: Platelet derived growth factor
VSMC: Vascular smooth muscle cells
